# Cognitive Dysfunction in Insomnia Phenotypes: Further Evidence for Different Disorders

**DOI:** 10.3389/fpsyt.2021.688672

**Published:** 2021-07-19

**Authors:** Michelle Olaithe, Melissa Ree, Nigel McArdle, Sara Donaldson, Maria Pushpanathan, Peter R. Eastwood, Romola S. Bucks

**Affiliations:** ^1^School of Psychological Science, University of Western Australia, Perth, WA, Australia; ^2^Centre for Sleep Science, School of Human Sciences, University of Western Australia, Perth, WA, Australia; ^3^Department of Pulmonary Physiology and Sleep Medicine, West Australian Sleep Disorders Research Institute, Sir Charles Gairdner Hospital, Nedlands, WA, Australia; ^4^College of Medicine and Public Health, Flinders Health and Medical Research Institute, Flinders University, Bedford Park, SA, Australia; ^5^School of Population and Global Health, University of Western Australia, Perth, WA, Australia

**Keywords:** insomnia, neuropsychology, phenotypes, paradoxical, short-sleep, cognition

## Abstract

**Study Objectives:** To determine cognitive profiles in individuals with short sleep duration insomnia (SSDI) and normal sleep duration insomnia (NSDI; also, paradoxical insomnia), compared to healthy sleepers.

**Method:** Polysomnographic (PSG) and neuropsychological data were analysed from 902 community-based Raine Study participants aged 22 ± 0.6 years of whom 124 met criteria for insomnia (53 with NSDI and 71 with or SSDI) and 246 were classified as healthy with normal sleep (i.e., without insomnia or other sleep disorders). Measurements of self- report (attention and memory) and laboratory-assessed (attention, episodic memory, working memory, learning, and psychomotor function) cognition and mood, and PSG-based sleep stages (% total sleep time; %TST) were compared between these 3 groups.

**Results:** In comparison to the healthy sleeper group, both insomnia groups had poorer self-reported attention, memory, mood, and sleep, and poorer laboratory-assessed attention (inconsistency). The NSDI group had less consistent working memory reaction time than healthy-sleepers or those with SSDI. The SSDI group had more inconsistency in executive function (shifting), and showed greater %TST in stage N1 and N3, and less REM sleep than either healthy-sleepers or those with NSDI.

**Conclusions:** Individuals with NSDI demonstrated greater working memory inconsistency, despite no laboratory assessed sleep problems, implicating early signs of pathophysiology other than disturbed sleep. Those with SSDI demonstrated different sleep architecture, poorer attention (inconsistency), and greater executive function (inconsistency) compared to healthy-sleepers and those with NSDI, implicating sleep disturbance in the disease process of this phenotype.

## Introduction

Insomnia is a highly prevalent sleep disorder (1), characterised by self-reported dissatisfaction with sleep quality or quantity, frequently expressed as difficulty initiating or maintaining sleep, or experiencing non-restorative sleep over many days, and accompanied by significant distress or daytime impairment (2). Risk factors for developing insomnia include female gender, older age, and chronic illness or pain (3). Comorbidities include other sleep disorders and psychiatric disorders, the latter being present in ~40% (1), obesity and metabolic problems (4). Consequences include an increased risk of accidents, poorer work productivity, higher pain levels, and more emotional and mental health problems (5). Cognitive problems are often found using laboratory-based computerised neuropsychological tests in individuals with insomnia (6, 7).

In a recent systematic review and meta-analysis of insomnia and cognitive performance, Wardle-Pinkston et al. (6) reported small to medium differences in the cognitive domains of complex attention, working memory, episodic memory, and executive function between individuals with and without insomnia. An earlier meta-analysis by Fortier-Brochu et al. (8) also reported small to medium effects for episodic memory, working memory, and executive functions (problem solving), whilst finding no differences in attention. Further, an investigation by Ballesio et al. (9), focussing on executive functions, found small effects for reaction times, but not accuracy, in the subdomains of inhibition, flexibility, and working memory.

The findings of these three papers contrast with those from a meta-analysis by Fulda and Shulz (10) who reported no differences in working memory, episodic memory, or attention, but did find contrasting ability between those with and without insomnia in a different aspect of executive functioning (generativity). Taken together, these reviews indicate that insomnia is associated with small to moderate, but variable, effects on cognition within the domains of working memory, episodic memory, and executive function.

Such inconsistencies may result from three possibilities: (1) Treating insomnia as a homogenous disorder, when it is not; (2) Assessing cognition using measures that are not sensitive to subtle changes in cognition, and/or; (3) Using older samples where age and comorbidity may confound the results. The review by Wardle-Pinkston et al. proposed that results may be variable due to differences in lab-assessed (i.e., objective) sleep factors between individuals with insomnia and psychometric testing sensitivity (6). These concepts are expanded upon below.

Two different phenotypes of insomnia are consistently identified: insomnia with short sleep duration (SSDI) and insomnia with normal sleep duration (NSDI; also called paradoxical insomnia or sleep state misperception) (11). These two phenotypes have different daytime symptoms (12), nocturnal symptoms (13), self-reported cognitive problems (14, 15), and underlying biology (16), all of which may also be associated with dissimilar objective neurocognitive challenges.

Individuals with SSDI have a short sleep period, and can accurately self-report wakefulness in the presence of lab-assessed wakefulness (13). They experience daytime fatigue, and self-reported problems with attention and memory (13). Further, SSDI is associated with self-reported and lab-assessed cognitive difficulties, mood disruption, physiological hyperarousal, and a higher risk of hypertension, diabetes, and all-cause mortality (17). SSDI also appears to be a biological marker of genetic predisposition to chronic insomnia (17).

In contrast, people with NSDI report short sleep time whilst lab-assessments indicate normal sleep time, and report being awake when PSG indicates sleep (16), termed sleep state-misperception. NSDI is also associated with self-reported and objective cognitive difficulties, mood disruption, and cortical hyperarousal (17). The Default Mode Network (DMN), is associated with self-referential information processing and has been shown to remain active in patients with insomnia, when it would deactivate in a healthy sleeper (16, 18, 19). Problems with the DMN are thought to result in deficits in self-referential and goal-directed behaviours (i.e., executive functions) (20), and have been implicated in mood disorders (12) and the development of dementia (15). This process may underlie the paradoxical experience of feeling awake while biologically asleep in those with NSDI (21).

Further, some of the inconsistent findings across reviews may be due to using measures of cognition that are not sensitive to small and/or early changes. All reviews [bar Fulda and Shulz, (10)] report small to moderate effects across papers, and propose that cognitive test sensitivity may account for differences across studies. To-date, the studies examining cognition in OSA have utilised traditional measures of accuracy and mean reaction time. None has explored intra-individual variability, which may be a more sensitive measure of cognitive performance. A measure of intra-individual variability, inconsistency, refers to intra-individual, short-term fluctuations in performance across trials, within a task, and can be measured using the intra-individual standard deviation (ISD) of reaction time on a trial-by-trial basis. Intra-individual variability has been demonstrated to be more sensitive than accuracy and mean reaction time to subtle cognitive changes, that may be exhibited in mild or early onset of disease (22, 23).

Finally, being middle-aged or older is itself associated with subtle decline in cognition and is frequently associated with greater comorbidity and disease burden (24–27). Declines in cognition are demonstrated in psychometric assessments using measures of reaction time, accuracy (frequently show an accuracy/speed trade-off), and in studies incorporating inconsistency measures (27). Given that the majority of studies of cognition in insomnia have recruited participants of middle to older age [e.g., in Wardle-Pinkston et al. (6) the mean age of participants across studies was 44.9 years], ageing effects in cognition may have confounded findings.

The present research aimed to contrast the cognitive function of individuals with SSDI and NSDI using self-report and computerised assessments providing measures of accuracy, speed, and inconsistency of performance, examining the cognitive constructs of attention, learning, working memory, executive function, and psychomotor funtion, in a sample of young adults.

We asked:

1. Do individuals with SSDI or NSDI differ from age-matched healthy sleeper controls from the same sample and, if yes, on what aspects of cognition?
2. How are the cognitive profiles of individuals with SSDI and NSDI different?
3. How are mood, daytime function outcomes, and sleep profiles of individuals with SSDI and NSDI different?

With regard to sleep, we hypothesised that: The SSDI group would have poorer self report and lab-assessed sleep than the NSDI and healthy sleepers, and; The NSDI group would have poor self-report but not lab assessed sleep than the healthy sleepers. Second, with regard to cognition, we hypothesised that: The NSDI and SSDI groups would report similar problems for self-report cognition, mood, and daytime function, and these would be greater than those reported by healthy sleepers, and; The SSDI group would show more extensive lab-assessed cognitive difficulties than the NSDI group.

## Materials and Methods

### Participants

The present analyses used data from the Raine Study, a multigenerational longitudinal epidemiological study established in 1989 (for more study details visit; rainestudy.org.au). Data from Generation 2 (Gen2) participants^1^ who completed the sleep study, actigraphy, and cognitive testing at the Gen2-22-year follow-up were used (*n* = 902).

Individuals with either a diagnosis of insomnia (DSM-5) or who met the insomnia criterion on the insomnia symptom questionnaire (ISQ; *n* = 156) were separated into NSDI (*n* = 63) or SSDI (*n* = 93) based on the Research Diagnostic Criteria (RDC) for PSG (28). For NSDI the RDC requires that PSG shows scored total sleep time (TST) ≥6.5 h, sleep efficiency (SE; TST/Time in bed × 100) ≥85%. For SSDI, the RDC requires short-sleep duration, PSG-based TST <6.5 h.

While participants were categorised by sleep study, they were retained if their self-report sleep diary data validated short or normal sleep duration: normal-sleep duration (*n* = 53*)* or short-sleep duration (*n* = 71). This was to ascertain if this single night of sleep under PSG was typical of an individual's sleep.

A healthy sleeper sample was constructed from those participants from the Raine Study who were without insomnia or other sleep disorders, had SE ≥85%, and TST ≥6.5 h on PSG (*n* = 324). Participants were retained if they reported normal sleep duration (TST>= 6.5 h) on their sleep diary (*n* = 246).

Participants who did not meet the insomnia selection criteria, healthy sleeper criteria, had a history of neurological, neurodevelopmental, significant psychiatric history, shift-work, or other sleep disorder/s (e.g., sleep apnoea with apnoea hypopnoea index≥15 and/or restless legs syndrome) were excluded (*n* = 532) from analyses.

**Figure 1:**
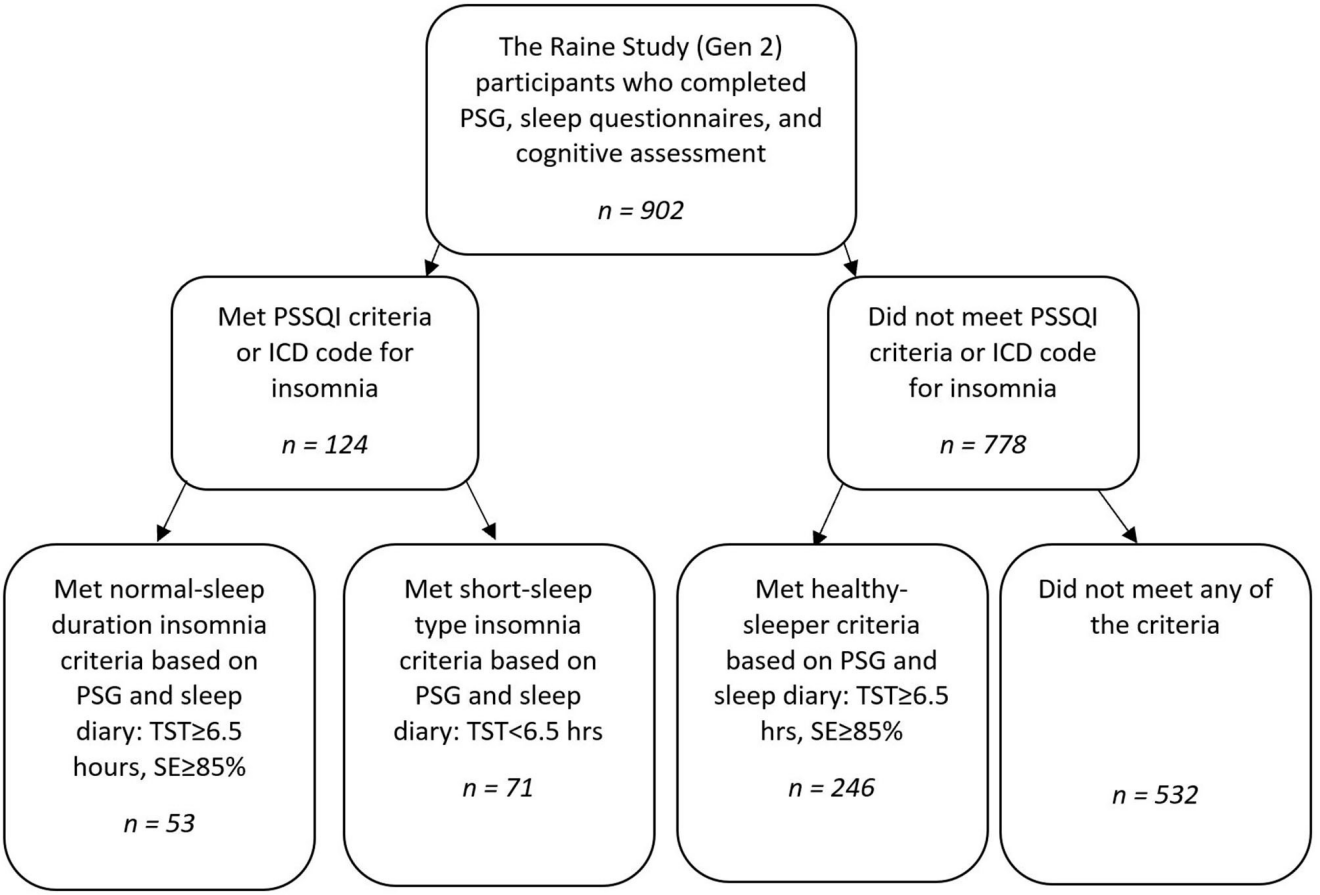
Flow chart of study group selection for NSDI, SSDI, and healthy controls.

A flow chart of study group selection is shown in Figure 1.

## Materials

### Sleep Study/PSG

Participants were administered full overnight Level 1 polysomnography (PSG) [Compumedics E-Series (Compumedics, Melbourne Australia)] and scored using Compumedics PSG 3 software at the Centre for Sleep Science, University of Western Australia. Equipment placement, sleep staging and event scoring was completed by experienced sleep technologists according to American Academy of Sleep Medicine criteria (29).

### Cogstate Computerised Battery

This computerised battery (30) provided tasks of attention (Card Identification), executive functioning (Set Shifting), learning (Continuous Paired Associates), psychomotor function (Detection Test), and working memory (One-back Task). The tasks show good correlations to traditional neuropsychological assessments (30) even when measured in populations that exhibit subtle cognitive changes (31). The Cogstate records both accuracy (whether the trial was answered correctly) and speed (time to make a correct response) on a trial-by-trial basis, allowing calculation of accuracy, mean response time, and variability in response time (inconsistency) using intra-individual standard deviations, for each task assessed.

To date, a few small sample studies examining cognition in obstructive sleep apnoea have included cognitive inconsistency amongst their measures. All have found greater IIV (i.e., more inconsistency or less cognitive stability) in those with obstructive sleep apnoea compared to healthy controls (32). IIV has not been reported in insomnia.

### Prospective and Retrospective Memory Questionnaire

A 16-item questionnaire (33) that provides a self-reported assessment of prospective and retrospective memory errors. The scale demonstrates good construct validity and internal reliability (α = 0.80–89) (33). Scores were generated for prospective and retrospective subscales.

### Attention-Related Cognitive Errors Scale

This 12-item questionnaire (34) provides a measure of everyday mistakes made when not paying sufficient attention to a task. The values are summed to give an overall score. ARCES scores are highly correlated with other scales assessing errors of sustained attention (35).

### Pittsburgh Sleep Symptom Questionnaire-Insomnia/Insomnia Symptom Questionnaire

A 13-item self-report tool (36) designed and used to identify insomnia symptoms and provide a case definition of insomnia. The items are consistent with the RDC (28) for insomnia and compared with interview methods of diagnosing insomnia the ISQ has good internal reliability (α = 0.89), moderate sensitivity (50–67%) and good specificity (91%) (36).

### Functional Outcomes of Sleep Questionnaire-10 (FOSQ-10)

This questionnaire (37) has 10-items designed to assess the impact of daytime sleepiness on daytime activity. The FOSQ-10 has good internal consistency (α = 0.87) and demonstrates changes over time with successful treatment of sleep disorders (38). Scores are reported for general productivity, vigilance, social outcomes, activity level, and sexual desire.

### Pittsburgh Sleep Quality Index

An 18-item questionnaire (39) that is designed to assess sleep habits, disturbances, and daytime impairments. This scale shows good internal consistency (α = 0.73) and shows a strong correlation with other scales assessing daytime function (40).

### Epworth Sleepiness Scale

This self-administered 8-item scale (41) assesses sleepiness in several every-day situations. The questionnaire has good internal consistency (α = 0.73–0.90). Excessive sleepiness is a score of 10 or more.

### Depression Anxiety and Stress Questionnaire-21 Item

This well-established 21-item questionnaire (42) assesses symptoms of depression, anxiety, and stress. It has demonstrated good internal reliability (α = 0.82–0.97) (42) and is an appropriate mood measure for sleep disordered populations, as it does not contain sleep-related items (43). Scores were generated for depression, anxiety, and stress subscales.

### Procedure

Neuropsychological testing and questionnaires were administered to all participants the night of or the morning after their sleep study at the Centre for Sleep Science. There was no adaptation night for this sleep study, hence we examined if this night was representative of an individual's habitual sleep against a weeklong sleep diary.

### Analysis

Descriptive statistics for the SSDI, NSDI, and healthy-sleeper groups are presented in Table 1.

**Table 1:** Descriptive statistics and lab-assessed sleep detail (PSG staged sleep; Stage as % of total sleep time) for the healthy sleepers, those with NSDI, and SSDI.

	**Healthy-sleep (*n* = 246)**	**NSDI (*n* = 53)**	**SSDI (*n* = 71)**
**Descriptive statistics**			
- Gender male n (%)	130 (48.1%)	15 (31.9%)	32 (34.4%)
- Age M ± SD (yrs)	22.2 ± 0.6	22.1 ± 0.6	22.2 ± 0.6
- BMI	22.0 ± 4.8	21.5 ± 4.5	21.3 ± 5.1
- Smoker (%)	28 (10.3%)	11 (20.0%)	18 (17.3%)
- ESS (total)	5.5 ± 0.1	8.9 ± 3.7	7.3 ± 3.8
- PSQI (total)	3.8 ± 1.6	6.1 ± 2.2	8.1 ± 3.2
**Lab-assessed sleep detail**			
- TST (hrs)	7.1 ± 0.4	7.3 ± 0.4	5.7 ± 0.8
- SE (%)	99.6 ± 0.6	100.0 ± 0.0	43.0 ± 5.0
- N1 (%TST)	8.41 ± 4.28	7.25 ± 3.22^∧^	10.14 ± 5.02^*^
- N2 (%TST)	46.73 ± 7.24	47.04 ± 7.35	44.94 ± 8.19
- N3 (%TST)	25.35 ± 5.04	25.45 ± 7.22^∧^	29.24 ± 9.17^*^
- REM (%TST)	19.50 ± 5.89	20.26 ± 5.19^∧^	15.68 ± 6.20^*^

*Effect sizes are reported in text for significant differences. TST, total sleep time; SE, sleep efficiency; ESS, Epworth sleepiness scale; PSQI, Pittsburgh sleep quality index; TST and SE values are taken from overnight sleep study*;

* *indicates that the marked group showed a significant difference to the healthy sleepers group*.

∧ *indicates a significant difference between the NSDI and SSDI groups*.

Residuals of the dependent variables were checked for and approximated normality as assessed through visual inspection of normality plots, p-p plots, and metrics of skew and kurtosis. Due to the differences in sample size, homogeneity of variance was assessed and was not violated. Independence of observations was met as all participants were counted in only one group (healthy, long-sleep duration, or short-sleep).

MANOVAs were conducted to compare the effect of group (normal-sleep duration insomnia, short-sleep type insomnia, and healthy sleepers) on lab-assessed sleep differences (PSG—-percentage N1, N2, N3, and REM), lab-based (Cogstate) and self-report cognition (retrospective and prospective memory subscales of the PRMQ and ARCES total score), self-reported mood (DASS-21- stress, anxiety and depression subscale scores), and self-reported daytime function (FOSQ—-general productivity, vigilance, social outcomes, activity level and sexual desire subscale scores). Lab-based (Cogstate) domains of cognition examined were attention, executive functioning, working memory, learning, and psychomotor function. For all domains, tests of accuracy, speed, and inconsistency of performance were assessed for group differences. Where tests of sphericity were violated an adjusted *F* value is reported. An alpha level of 0.05 was used, except where omnibus group interaction or main effect differences were found, then *post-hoc* Bonferroni-corrected tests were conducted (0.05 × number of tests).

## Results

Overall, these data show that the cohort was relatively young (22-yrs), that lab-assessed sleep efficiency was poor in those with SSDI, and that both phenotypes reported high levels of sleepiness, and low levels of sleep quality (as per cut-offs on the PSQI).

### Lab-Assessed Sleep Differences

Means for sleep study data are presented in Table 1.

Interactions were significant for group by sleep stage, Roy's Largest Root, *F*_(3, 427)_ = 2267.69, *p* < 0.001. Indicating that time spent in sleep stages varied by group.

*Post-hoc* comparisons indicated that participants with SSDI spent a larger percentage of TST in N1 compared to the healthy sleepers [*p* < 0.001; Cohen's *d* = 0.39 (95% CI, LL = 0.12, UL = 0.65)] and to those with NSDI [*p* < 0.001; Cohen's *d* = 0.67 (95% CI, LL = 0.31, UL = 1.02)]. Those with SSDI also spent a higher percentage of TST in N3 than those with healthy sleep [*p* < 0.001; Cohen's d = 0.63 (95% CI, LL = 0.36, UL = 0.89)] or with NSDI [*p* < 0.001; Cohen's *d* = 0.45 (95% CI, LL = 0.09, UL = 0.81)]. Finally, those with SSDI had lower %REM than healthy sleep [*p* < 0.001; Cohen's *d* = 0.72 (95% CI, LL = 0.46, UL = 0.99)] and NSDI [*p* < 0.001; Cohen's *d* = 0.88 (95% CI, LL = 0.52, UL = 1.23)].

In summary, participants with SSDI demonstrated a greater percentage of their total sleep time in NREM sleep and less time in REM sleep than the healthy sleepers or those with NSDI.

### Lab-Assessed Cognition

Interactions were not significant for accuracy or reaction time, however, a significant group by cognitive test interaction was found for inconsistency, Roy's Largest Root, *F*_(8, 1212)_ = 3.15, *p* = 0.002, revealing a different pattern of cognitive performance for each group.

For attention (inconsistency) the healthy sleeper group showed more consistent response times than the NSDI [*p* < 0.001; Cohen's *d* = 0.91 (95% CI, LL = 0.61, UL = 1.20)] and the SSDI (*p* = 0.006; Cohen's *d* = 0.44 (95% CI, LL = 0.18, UL = 0.71) groups. For working memory (inconsistency), the healthy sleepers had more consistent response times than the NSDI [*p* = 0.005; Cohen's *d* = 0.31 (95% CI, LL = 0.01, UL = 0.61)] but not the SSDI group. For executive function, shifting (inconsistency) healthy sleepers were more consistent in their response times than the SSDI group [*p* < 0.001; Cohen's *d* = 0.55 (95% CI, LL = 0.29, UL = 0.82)], but not the NSDI. Means for all lab-assessed cognitive tests are presented in Table 2.

**Table 2:** Means and standard deviations for lab-assessed cognitive assessments (CogState) for the healthy sleepers, those with NSDI, and SSDI.

	**Healthy-sleep (*n* = 246)**	**NSDI (*n* = 53)**	**SSDI (*n* = 71)**
**Attention (card identification: IDN)**			
- Accuracy	1.35 ± 0.17	1.31 ± 0.27	1.38 ± 0.12
- Speed	2.63 ± 0.06	2.64 ± 0.08	2.65 ± 0.07
- Consistency	0.07 ± 0.02	0.09 ± 0.03^*^	0.08 ± 0.03^*^
**Executive function (set-shifting: SETS)**			
- Accuracy	1.11 ± 0.12	1.11 ± 0.12	1.09 ± 0.11
- Speed	2.72 ± 0.17	2.73 ± 0.17	2.73 ± 0.02
- Consistency	0.32 ± 0.07	0.34 ± 0.11	0.36 ± 0.08^*^
**Learning (continuous-paired: CPAL)**			
- Accuracy	1.22 ± 0.26	1.26 ± 0.23	1.23 ± 0.26
- Speed	3.22 ± 0.14	3.23 ± 0.13	3.21 ± 0.12
- Consistency	0.89 ± 0.23	0.87 ± 0.24	0.90 ± 0.22
**Psychomotor function (detection test: DET)**			
- Accuracy	1.41 ± 0.31	1.40 ± 0.28	1.43 ± 0.03
- Speed	2.45 ± 0.08	2.44 ± 0.09	2.45 ± 0.09
- Consistency	0.07 ± 0.04	0.08 ± 0.04	0.08 ± 0.04
**Working memory (one-back task: ONB)**			
- Accuracy	1.23 ± 0.26	1.25 ± 0.27	1.23 ± 0.25
- Speed	2.80 ± 0.09	2.81 ± 0.13	2.81 ± 0.09
- Consistency	0.12 ± 0.03	0.13 ± 0.04^*^	0.13 ± 0.03

*Effect sizes are reported in text for significant differences. Higher inconsistency scores indicate more inconsistent/poorer performance. Higher speed scores indicate slower/poorer performance. Higher accuracy scores indicate higher/better performance*;

* *indicates that the marked group showed poorer performance than the healthy sleepers group*.

There were no group differences on psychomotor function or learning, nor any interactions with group.

Taken together, these results indicate that the healthy participant group experienced better lab-assessed cognition overall and that both insomnia groups demonstrated greater inconsistency; the NSDI group demonstrated poorer working memory than the healthy sleepers; and, the SSDI group demonstrated poorer executive function, shifting, than the healthy sleepers.

### Self-Report Cognition

Interactions were significant for group by self-report cognitive test, Roy's largest root, *F*_(3, 423)_ = 11.29, *p* < 0.001.

*Post-hoc* comparisons indicated that all mean scores for all self-reported cognition assessments for the healthy sleeper group were significantly better than the NSDI (*p* < 0.001) and SSDI groups (*p* < 0.001), however the insomnia groups did not differ. Means are presented in Table 3.

**Table 3:** Means and standard deviations for the self-reported cognition assessments (PRMQ and ARCES) for the healthy sleepers, those with NSDI, and SSDI.

	**Healthy-sleep (*n* = 246)**	**NSDI (*n* = 53)**	**Cohen's d, 95% CI (L,U)**	**SSDI (*n* = 71)**	**Cohen's d, 95% CI (L,U)**
PRMQ total scale	33.74 ± 8.01	41.07 ± 9.70^*^	0.88 (0.58, 1.18)	40.04 ± 9.95^*^	0.74 (0.48, 1.01)
- Prospective memory subscale	18.10 ± 4.54	22.24 ± 5.16^*^	0.89 (0.59, 1.19)	21.64 ± 5.47^*^	0.74 (0.48, 1.01)
-Retrospective memory subscale	15.64 ± 4.03	18.82 ± 5.16^*^	0.75 (0.45, 1.28)	18.54 ± 5.14^*^	0.67 (0.41, 0.94)
ARCES	30.21 ± 6.27	36.64 ± 7.67^*^	0.98 (0.69, 1.28)	35.98 ± 7.32^*^	0.89 (0.62, 1.15)

* *indicates that the marked group showed a significant difference to the healthy sleepers group. Self-report shows no difference in performance between SSDI and NSDI sleepers. Subscale scores, prospective and retrospective memory scores were used in the repeated measures ANOVA*.

These results indicate that people from both the SSDI and NSDI groups reported poorer attention, retrospective, and prospective memory than individuals with healthy sleep, and that the insomnia groups did not differ from one another.

### Self-Reported Mood and Daytime Function

Interactions were significant for group by mood [Roy's Largest Root: *F*_(3, 402)_ = 5.868, *p* < 0.001] and daytime function by group [Roy's Largest Root: *F*_(5, 391)_ = 26.596, *p* < 0.001].

*Post-hoc* comparisons indicated that mean scores for all assessments of self-reported mood (*p* < *0.0*01) and functional sleep outcomes (*p* < *0.0*01, with the exception of the FOSQ vigilance subscale, *p* = *0.0*03) were significantly higher in both insomnia groups than in healthy sleepers, though the insomnia groups did not differ. Means are presented in Table 4.

**Table 4:** Means and standard deviations for self-reported mood and daytime function assessments (DASS-21 and FOSQ questionnaires) for the healthy sleepers, those with NSDI, and SSDI.

	**Healthy-sleep (*n* = 246)**	**NSDI (*n* = 53)**	**Cohen's d, 95% CI (L,U)**	**SSDI (*n* = 71)**	**Cohen's d, 95% CI (L,U)**
DASS-21 total scale	14.46 ± 13.45	42.41 ± 26.32	1.70 (1.40, 2.00)	40.03 ± 23.89	1.56 (1.30, 1.83)
- Depression subscale	4.24 ± 6.11	13.86 ± 10.57^*^	1.36 (1.06, 1.65)	13.33 ± 9.57^*^	1.29 (1.03, 1.56)
- Anxiety subscale	3.15 ± 4.03	10.23 ± 8.55^*^	1.38 (1.09, 1.68)	9.38 ± 7.87^*^	1.21 (0.95, 1.48)
- Stress subscale	6.40 ± 5.93	16.98 ± 10.40^*^	1.53 (1.23, 1.83)	17.50 ± 9.88^*^	1.59 (1.32, 1.85)
FOSQ total scale	18.00 ± 1.60	15.37 ± 2.63	1.44 (1.14, 1.74)	15.72 ± 2.41	1.26 (0.99, 1.52)
- General productivity subscale	3.45 ± 0.53	2.72 ± 0.78^*^	1.26 (0.96, 1.55)	2.88 ± 0.75^*^	0.97 (0.71, 1.24)
- Vigilance subscale	3.59 ± 0.43	3.20 ± 0.60^*^	0.84 (0.54, 1.14)	3.37 ± 0.60^*^	0.47 (0.20, 0.73)
- Social outcomes subscale	3.84 ± 0.02	3.46 ± 0.71^*^	1.28 (0.98, 1.57)	3.46 ± 0.10^*^	0.75 (0.72, 0.78)
- Activity level subscale	3.49 ± 0.44	2.84 ± 0.72^*^	1.30 (1.00, 1.60)	2.83 ± 0.61^*^	1.37 (1.10, 1.63)
- Sexual desire subscale	3.64 ± 0.57	3.11 ± 0.84^*^	0.85 (0.55, 1.15)	3.29 ± 0.83^*^	0.55 (0.28, 0.81)

* *indicates that the marked group showed poorer performance than the healthy sleeper group. Self-report shows no difference in performance between SSDI and NSDI sleepers. Subscale scores, depression, anxiety and stress scores, were used in the repeated measures ANOVA for mood, and subscale scores for general productivity, vigilance, social outcomes, activity level, and sexual desire were used in the repeated measures ANOVA for daytime function*.

These results suggest that both the NSDI and SSDI groups report poorer mood across higher depression, stress, and anxiety, and report that their poor sleep impacts on their ability to function day-to-day, with regards to productivity, vigilance, social outcomes, activity levels, and sexual desire, than healthy sleepers.

## Discussion

**Figure 2:**
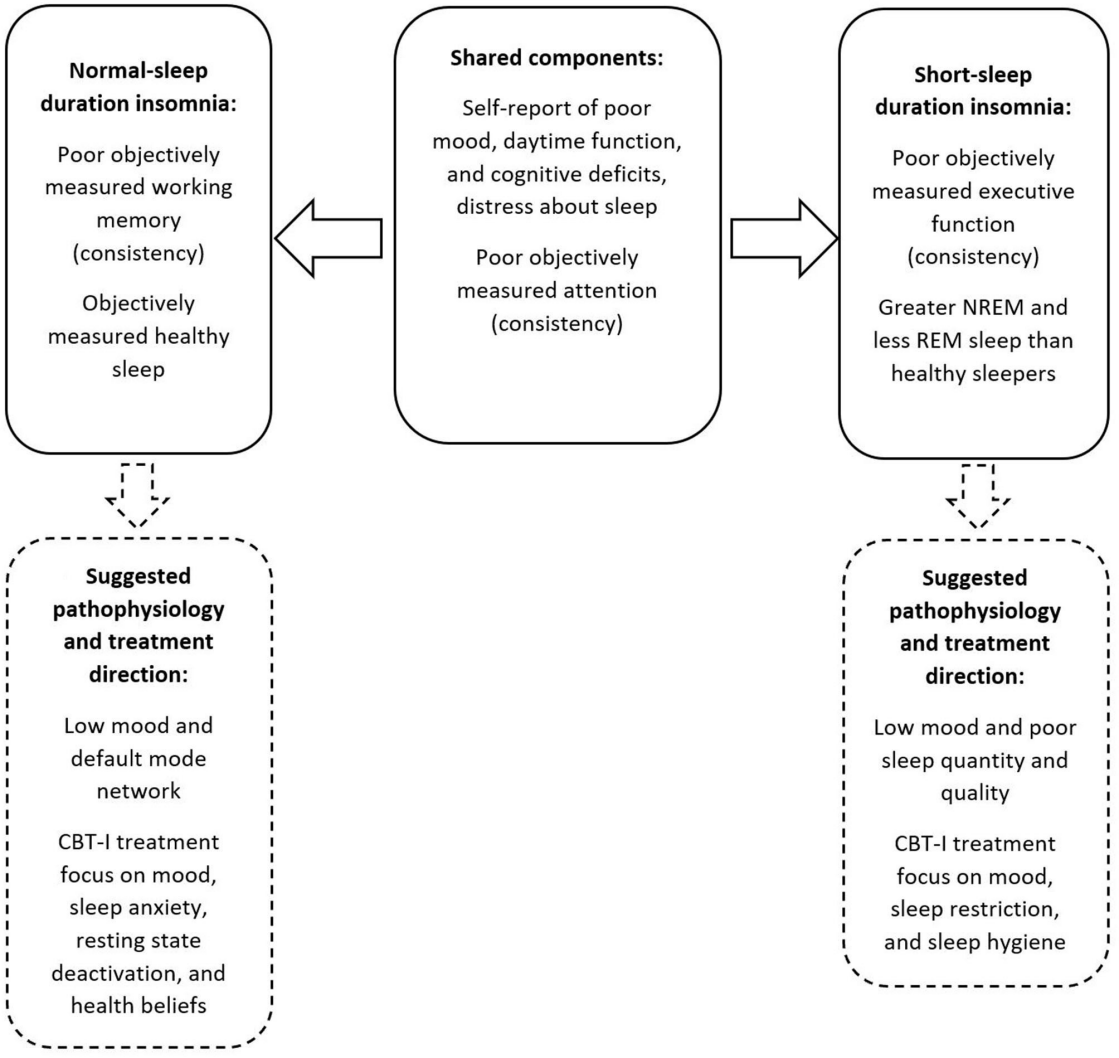
Summary of the shared and separable profiles of SSDI and NSDI, and suggested pathophysiology and treatment focus.

This paper aimed to characterise the two main insomnia phenotypes (SSDI and NSDI) with detailed lab and self-report cognitive and mood assessments, in a large sample of Gen2 participants from the Raine Study. The results demonstrate that those with SSDI and NSDI self-report problems with attention and memory, daytime function due to poor sleep. Further, those with SSDI and NSDI show greater inconsistency in performance on objective attention tasks. Those with SSDI show less consistent executive functioning and those with NSDI show less consistent working memory, than those with healthy sleep. Further, those with SSDI demonstrated sleep architecture that was different from NSDI and healthy sleepers, while those with NSDI showed relatively healthy lab-assessed sleep. These differences are summarised in Figure 2.

### Self-Reported Mood, Function, and Sleep

In line with the literature (1, 6, 13), those with both SSDI and NSDI self-report problems with cognition (attention and memory), daytime function, mood, and sleep quality. As self-reported sleep quality, daytime function, and mood are core components of an insomnia diagnosis (2), this result is not surprising. Further, these findings suggest that SSDI and NSDI do not differ in terms of self-report measures of cognition, however they do differ on objective assessments.

### Lab-Assessed Cognition and Sleep

Both phenotypes of insomnia exhibit inconsistency in attention. Whilst Wardle-Pinkston et al. (6) also reported attention problems in insomnia in their meta-analysis, Fulda and Schulz (10) and Fortier Brochu et al. (8) did not, but all three studies reported accuracy. This sample, using a younger sample than previous studies, found no evidence of problems with attention accuracy, but did find greater inconsistency in the speed of responding to attention trials in insomnia. Likewise, whilst past studies, also with older participants, have reported deficits in working memory and executive function accuracy (6, 8, 10), we found more subtle effects in inconsistency of responding in these domains. These differences were varied across the insomnia phenotypes: those with NSDI were less consistent in working memory despite relatively healthy sleep, while those with SSDI were less consistent in executive functioning, and had different sleep architecture (more N1 and N3, and less REM, as a percentage of total sleep time) than healthy sleepers and those with NSDI.

A high amount of N3 sleep, seen here in those with SSDI, has been noted to indicate rebound sleep. This is considered ‘recovery sleep’ as shown after sleep deprivation or chronic sleep restriction (44). This supports the diagnosis of SSDI, provided by PSG, diary, and self-report symptoms in this study, and supports the idea that the cognitive problems shown in SSDI are the result of chronic sleep loss. Conversely, those with NSDI showed subtle objective and self-reported cognitive deficits, despite no evidence of lab-based sleep loss.

That separable cognitive profiles and sleep profiles were demonstrated for the different phenotypes suggests future directions for providing a differential diagnosis. Currently, as in this paper, overnight sleep study or actigraphy are used to assess the mismatch between self-report and lab-assessed sleep and distinguish short- from normal normal-sleep duration insomnia (28). However, sleep studies, actigraphy, and full neuropsychological assessment can be expensive and time consuming, and are activities requiring a high degree of specialised training. The present paper suggests that cognitive tasks such as those used here, may provide further information to profile those with insomnia. When computerised, these tasks are relatively quick (7-min in healthy participants), and easy to administer. However, these results require replication by other groups and the ability of these differences to discriminate groups requires validation.

Working memory and executive functions are separable but related components of cognition. Working memory is a limited capacity cognitive system that can hold information ready for processing for a limited time (45). The executive function factor assessed here, shifting, is related to working memory, as it assists with shifting attentional control quickly (46). Other aspects of executive function [updating, generativity, fluid problem solving, and inhibition (46, 47)] were not assessed in the present paper, and as such we have an incomplete picture of executive function in these subtypes of insomnia. A complete examination of executive functions in insomnia phenotypes will provide greater understanding of how executive functions are impacted and deepen our understanding of different cognitive profiles in insomnia.

There were no psychomotor or learning problems for either phenotype uncovered in these analyses. These findings are in line with past studies of psychomotor function (6, 8, 10) and in contrast to past explorations of learning in insomnia (6). Learning, in the current sample, may not have been problematic as Generation-2 from the Raine Study were young (Age, M = 22 years, at the time of assessment). Young age is protective for cognition. Wardle-Pinkston et al. (6) investigated age as a moderator in their analyses showing that older age was associated with larger effect sizes in the differences between healthy sleepers and individuals with insomnia. To explore the impact of age, future research could investigate phenotypic differences in the progression of cognitive deficits in longitudinal datasets, or compare cognitive function in older and younger samples. The Raine Study will make an excellent space to explore this concept as data continue to be collected on the same individuals, their parents (Generation 1), their grandparents, (Generation 0) and now the children of Generation 2 (i.e., Generation 3).

Where there were cognitive differences, for both phenotypes of insomnia these were in maintaining consistent response times throughout a testing trial, whilst accuracy and speed were not impacted (6, 8, 10). This finding, taken together with the small effects evidenced in meta-analyses and inconsistent findings across the field, identifies a need for sensitive measures of cognition that capture moment-to-moment performance stability, such as intra-individual variability (IIV) (48). Previous studies have not examined inconsistency, reporting only measures of accuracy and speed, meaning it is possible these consistency differences were present in earlier samples. The literature on IIV indicates it is a sensitive measure of early cognitive change and is predictive of later cognitive dysfunction and decline (49). Future follow-up assessments of the Raine Study participants will be able to track those showing early cognitive instability to see if clearer cognitive problems, in accuracy, speed, and/or a wider set of cognitive domains, develop.

Further, the present sample were relatively healthy, young individuals involved in research from before birth to their mid-twenties, possibly leading to selection bias. As comorbidity, including overweight and psychological diagnoses, can independently impact sleep, sleep disorders, and cognition (50), future studies may wish to investigate the interaction of comorbidity and/or other demographic features with cognition in those with insomnia.

### Pathophysiology

Hyperarousal is a core pathophysiological feature of insomnia, in general, and is explained in two different models: a psychological model and a physiological model. The psychological model posits that worry and rumination about life stress, and about sleep itself, disrupt sleep, whereas, the physiological model posits that hyperarousal is due to a higher level of neuroendocrine and metabolic functioning, which disrupts sleep.

While it is possible that cognition and sleep, in both insomnia phenotypes, are impacted by psychological processes, as certainly belief impacts biological health in other areas, including stress (51) and treatment uptake (52, 53), such a model, with one road to pathology, does not explain the different cognitive and sleep profiles of these disorders, as evidenced here. There is some discussion in the literature of differing types of hyperarousal across the two phenotypes. Short sleep is associated with physiological hyperarousal while NSDI is associated with cortical hyperarousal (17). While physiological hyperarousal is a heightened stress state due to negative thoughts, cortical hyperarousal is increased activation of the reticular formation causing an increase in wakefulness. The result from the present paper provide further evidence of two different disorders that may have different underlying causes of hyperarousal.

### Short-Sleep Duration Insomnia

Disrupted sleep appears to be a core feature of short-sleep duration insomnia, with less total time asleep, a higher % of NREM, and lower percent of REM sleep, when compared to those with healthy sleep and NSDI. This pattern of sleep architecture is similar to that seen in attention deficit hyperactivity disorder (ADHD) (54), another disorder of hyperarousal. This suggests potential for some shared pathophysiology, for example, problems in the dorsolateral pons, an area of the brain implicated in modulating arousal and sleep states (55, 56). This biological basis for poor sleep may then impact mood and thinking, and then move into a more cyclic relationship between these features.

Further, short sleep duration is associated with chronic insomnia (17). As chronicity in many other disorders is associated with poorer health and cognitive outcomes (24), it is surprising that the results of the present paper do not indicate more severe cognitive problems for those with SSDI. However, the sample was young and relatively healthy, and as cognitive problems were only witnessed in inconsistency (an indicator of early cognitive change), it is possible these individuals have not had sufficient exposure to insomnia, and/or that young age may provide a “buffer” for cognitive problems. For example, N3 declines with age, whereas in the present sample, there was greater quantity of N3 sleep, perhaps reflecting a homeostatic way of compensating for sleep loss that may not be present in an older sample. Examination of the impact of chronicity among these two phenotypes is an important future direction.

### Normal-Sleep Duration Insomnia

By contrast, poor lab-assessed sleep is not a core feature of NSDI, despite less consistent response times to cognitive tasks in comparison to healthy sleepers. This suggests that something else is affecting cognition and mood than the sleep disruption seen in SSDI.

The DMN is a network of interacting brain regions that is active when a person is at rest (18) and is thought to be responsible for “off-line” cognitive functions. This network has been implicated in the paradoxical experience of feeling awake while lab-assessed assessments detect sleep, and has been purported to be responsible for cognitive problems in other neurological disorders, including schizophrenia (57), where there are also working memory deficits and problems with self-referential thoughts (58). As such, NSDI appears not to be a sleep disorder *per se*, rather a disorder of neural networks.

### Impact on Diagnosis and Treatment

Different pathophysiology, as suggested by these results, directly impacts the most appropriate therapy and suggests a need for early differential diagnosis.

Cognitive behavioural therapy for insomnia (CBTi) is considered a first-line treatment for insomnia with results superior to benzodiazepines (59). Among other aspects, CBTi includes core components to address unhelpful health beliefs, low mood, and unconsolidated sleep. These factors are important modifiable precipitating and maintaining features of insomnia. While CBTi demonstrates good results, not all patients receive benefits from this treatment (59). It is plausible that those who do not benefit are those for whom the underlying cause has not been addressed. For example, if short-sleep duration is due to biologically-based hyperarousal then CBTi may have limited ability to address such predisposing factors.

Further work to understand these potentially different disorders and their pathophysiology is crucial to inform therapy and provide more individualised treatments.

## Data Availability Statement

The datasets presented in this article are not readily available as the data belong to the Raine Study. Requests to access this data can be made to this administering institution. Requests to access the datasets should be directed to https://rainestudy.org.au/.

## Ethics Statement

The studies involving human participants were reviewed and approved by the Human Ethics Committee, University of Western Australia. The patients/participants provided their written informed consent to participate in this study.

## Author Contributions

All authors listed have made a substantial, direct and intellectual contribution to the work, and approved it for publication.

## Conflict of Interest

The authors declare that the research was conducted in the absence of any commercial or financial relationships that could be construed as a potential conflict of interest.

## Footnotes

^1^The Raine Study index participants, who were born into the study between 1990 and 1991.
